# Artificial Intelligence–Based Chatbot for Anxiety and Depression in University Students: Pilot Randomized Controlled Trial

**DOI:** 10.2196/20678

**Published:** 2021-08-12

**Authors:** Maria Carolina Klos, Milagros Escoredo, Angela Joerin, Viviana Noemí Lemos, Michiel Rauws, Eduardo L Bunge

**Affiliations:** 1 Interdisciplinary Center for Research in Health and Behavioral Sciences (CIICSAC), Universidad Adventista del Plata (UAP). National Scientific and Technical Research Council (CONICET). Libertador San Martín, Entre Ríos Argentina; 2 X2AI inc. San Francisco, CA United States; 3 Department of Psychology Palo Alto University Palo Alto, CA United States

**Keywords:** artificial intelligence, chatbots, conversational agents, mental health, anxiety, depression, college students

## Abstract

**Background:**

Artificial intelligence–based chatbots are emerging as instruments of psychological intervention; however, no relevant studies have been reported in Latin America.

**Objective:**

The objective of the present study was to evaluate the viability, acceptability, and potential impact of using Tess, a chatbot, for examining symptoms of depression and anxiety in university students.

**Methods:**

This was a pilot randomized controlled trial. The experimental condition used Tess for 8 weeks, and the control condition was assigned to a psychoeducation book on depression. Comparisons were conducted using Mann-Whitney *U* and Wilcoxon tests for depressive symptoms, and independent and paired sample *t* tests to analyze anxiety symptoms.

**Results:**

The initial sample consisted of 181 Argentinian college students (158, 87.2% female) aged 18 to 33. Data at week 8 were provided by 39 out of the 99 (39%) participants in the experimental condition and 34 out of the 82 (41%) in the control group. On an average, 472 (SD 249.52) messages were exchanged, with 116 (SD 73.87) of the messages sent from the users in response to Tess. A higher number of messages exchanged with Tess was associated with positive feedback (*F*_2,36_=4.37; *P*=.02). No significant differences between the experimental and control groups were found from the baseline to week 8 for depressive and anxiety symptoms. However, significant intragroup differences demonstrated that the experimental group showed a significant decrease in anxiety symptoms; no such differences were observed for the control group. Further, no significant intragroup differences were found for depressive symptoms.

**Conclusions:**

The students spent a considerable amount of time exchanging messages with Tess and positive feedback was associated with a higher number of messages exchanged. The initial results show promising evidence for the usability and acceptability of Tess in the Argentinian population. Research on chatbots is still in its initial stages and further research is needed.

## Introduction

The most prevalent disorders in Argentina are anxiety (16.4%) and mood (12.3%) disorders. The average age for the onset of these conditions is 20 years [1]. The Pan American Health Organization (PAHO) and the Argentinian Ministry of Health have highlighted the importance of optimizing health care services for individuals who are not receiving any form of psychological care [2]. Furthermore, the epidemiological data collected in Argentina emphasizes the need for strategies that prevent delays to treatment access [1]. Behavioral intervention technologies (BITs) are a novel and effective delivery format that can expand the mental health services offered and facilitate early access to those in need [3]. Chatbots are examples of BITs that represent an opportunity for addressing delays associated with access to treatment for depression and anxiety [4]. However, no studies on the use of chatbots for analyzing depression and anxiety have been conducted in Argentina.

Chatbots developed using artificial intelligence (AI) are emerging in the field of psychology [5]. Currently, there are two chatbots that have addressed anxiety and depressive symptoms, Woebot [6] and Tess [7]. Woebot is a chatbot based on the cognitive behavioral approach with evidence for the reduction of anxiety and depressive symptoms in students during a follow-up after 2 weeks. Fulmer et al [7] reported a reduction in depressive and anxiety symptoms in college students using Tess, a chatbot that provides support and psychoeducation through an integrative approach. Although the research completed by Fulmer et al [7] and Fitzpatrick et al [6] reported decreased depressive and anxiety symptoms in college students, these studies were performed in the United States. To the best of our knowledge, there are no studies on chatbots used for addressing mental health disorders in Spanish-speaking populations. Other examples of chatbots with empirical support are Manage Your Life Online (MYLO) that focuses on problem solving [8]; Shim, for well-being based on the cognitive behavioral approach and elements of positive psychology [9]; Tess for pediatric obesity and prediabetes treatment [10]; and Wysa, a chatbot that uses cognitive behavioral therapy, behavioral reinforcement, and mindfulness techniques to support patients with depression [11]. Research studies on chatbots for mental health have several limitations such as small sample sizes and short-term follow-ups [6,7]. Additionally, current chatbots for mental health promotion present several problems, such as the lack of recognition of the emotional tone of users, crisis identification and management, as well as the need for strategies to reduce the frustration arising from feelings of incomprehension by users when the chatbot does not respond accurately.

The present study aims to assess the viability and acceptability of psychological interventions delivered through Tess to college students in Argentina. The objectives of this study were as follows: (1) identify participant flow from recruitment to follow-up; (2) understand aspects related to the usage patterns of Tess, such as the number of messages sent and exchanged; (3) examine the relationship between the feedback provided by the participants and the number of messages exchanged with Tess; and (4) compare the outcomes on depression and anxiety between and within groups among the college students who completed the study. Although the focus of this research was not the effectiveness of the chatbot, comparisons were made between the experimental and control groups to obtain preliminary data for future randomized controlled trials given the importance of obtaining preliminary information about the viability and acceptability of Tess as a means of psychological intervention for college students in Argentina.

## Methods

### Trial Design

This was a pilot randomized controlled parallel-group trial. The experimental group had access to Tess for 8 weeks and the control group to a psychoeducation electronic book.

### Participants

The participants were college students in Entre Ríos, Argentina. The inclusion criteria were as follows: being a resident of Argentina, 18 years or older, and a college student, as well as providing informed consent. Recruitment was conducted through presentations in different university courses. Participants who provided consent were assigned to the experimental or control group by simple randomization conducted through a Python algorithm.

### Intervention

#### Experimental Group

The experimental group utilized Tess, an AI-based chatbot that delivers brief text conversations as comprehensive support for mental health. Tess sends reminders, psychoeducational content, and emotional support responses based on what the users express. Tess combines words and emojis in the messages for providing a more user-friendly experience. Tess responds with prescribed statements to replicate empathetic answers that are appropriate for the emotion or concern expressed by the participants. For example, a participant expressing anxious feelings would be offered a relaxation strategy. The conversations offered by Tess were based on the cognitive behavioral model [12], emotion-focused therapy [13], solution-focused brief therapy [14], and motivational interviewing [15]. Such conversations were developed by mental health experts. After each conversation, Tess asked, “Was our conversation helpful?” If a user responded positively (eg, “yes, thank you”) to an intervention based on cognitive behavioral therapy (CBT) and negatively (eg, “no, not really”) to emotion-focused therapy, Tess would then offer more CBT-based interventions. For users who answered in a negative or neutral manner, Tess would offer alternative interventions.

In the present study, customized conversations for university students in Argentina were elaborated, revised, and tested within the framework of a previous study developed in the United States [7]. During the 8-week intervention for this test, Tess initiated contact asking about the emotions and moods of the participants once a day during the initial weeks and every other day in the following weeks. All the conversations with Tess occurred through Facebook messenger.

#### Control Group

An electronic psychoeducation book focusing on affective symptoms was provided to the participants in the control group [16]. The provided evidence-based information and resources helped students identify and seek treatment for depressive symptoms.

### Engagement and Feedback

Engagement was measured using the number of messages exchanged with Tess. In addition, the dropout rates in the experimental and control groups were analyzed. The perceived feedback of the participants was collected after each conversation with Tess through the following question: “Was our conversation helpful?” The answers from the users were coded as positive, negative, or ambivalent and assigned values of 1, 2, and 3, respectively. For instance, if a user responded saying “yes, thank you,” then that response was coded as positive.

### Measures

The Patient Health Questionnaire-9 (PHQ-9) [17] is a self-reporting questionnaire comprising 9 items that evaluate the frequency and severity of depressive symptoms during the last 2 weeks. Each of the 9 items is based on the *Diagnostic and Statistical Manual of Mental Disorders-IV* (*DSM-IV*) criteria, which are scored from 0 (not at all) to 3 (nearly every day). The PHQ-9 is one of the most used measures to assess depressive symptoms and has been validated in Argentina with adequate psychometric properties (Cronbach α=.87) [18]. The first 2 items are considered screening criteria (PHQ-2); if these are scored with 0 or 1, then an absence of symptoms is assumed. Scores ranging from 5 to 9 are interpreted as mild, from 10 to 14 as moderate, from 15 to 20 as moderately severe, and over 20 as severe.

The Generalized Anxiety Disorder Scale (GAD-7) [19] is a 7-item self-reporting scale that evaluates the frequency and severity of anxious thoughts and behaviors during the last 2 weeks. Items are based on the diagnostic criteria of the *DSM-IV* and scored from 0 (not at all) to 3 (nearly every day). Rodríguez de Behrends and Brenlla [20] reported an adequate reliability level (Cronbach α=.74) for the Argentinian population.

### Ethical Aspects

The study was approved by the Research Ethics Committee of the Faculty of Health Sciences (FCS) of the Universidad Adventista del Plata (UAP), National Registry of Health Research (RENIS, reference number: CE000237), and Ministerial Resolution of the Ministry of Health of the Province of Entre Ríos (reference number: 3999). This resolution is recorded in ACT 1-2019 of the registration of this committee. Participants expressed their consent in a form according to the personal data protection law (Argentine National Law 25.326) through checkbox selection (electronic signature) on a closed form.

Data were collected through Tess. All personally identifiable information was eliminated in the transcriptions downloaded from Tess. The downloaded data were processed and stored using secure servers and were compliant with the Health Insurance Portability and Accountability Act. Upon completion of the study, the control group obtained access to Tess for 8 weeks and both groups were granted free access to Tess for a year. If a participant expressed suicidal ideations, Tess was programmed to provide the National Line of Suicide Prevention numbers, the crisis text line, and 911, and encourage seeking professional help.

### Data Analysis

The data collected was entered and analyzed using the Statistical Package for the Social Sciences (Version 20.0; IBM Corporation) [21]. The number of messages exchanged was considered to assess the feasibility and acceptability of Tess. Additionally, the participants' qualitative feedback was analyzed by two researchers (CK and ME) and coded into three categories: positive, negative, and ambivalent. A data analysis protocol was carried out. The treatment of missing data through multiple imputation or plausibility analysis techniques was not possible owing to the high percentage of participants who dropped out of the intervention [22].

A one-factor analysis of variance (ANOVA) was applied to determine if feedback (positive, negative, or ambivalent) impacted the number of interactions that users had with Tess. To examine the baseline characteristics between samples, a *t* test was performed for independent samples to compare anxiety levels. The Mann-Whitney *U* statistic (respecting the ordinal nature of the variables; namely, if the first 2 items were scored 0 or 1, the system did not ask the subsequent items) was used to compare depressive symptoms.

To evaluate the effects between conditions, a *t* test was performed for the independent samples to assess the anxiety symptoms and the Mann-Whitney *U* statistic was used to compare the mean ranges of depression. To assess the longitudinal effects from the baseline to week 8 within conditions, a *t* test was performed for related samples assess the anxiety symptoms and the Wilcoxon test was performed to compare the mean ranges of depression. To complement the significance test, the effect sizes in the intragroup and intergroup tests were calculated. For the *t* tests, the effect size was calculated using Cohen *d*; measures between 0.2 and 0.3 were labeled “small effect,” around 0.5 as “moderate effect,” and above 0.8 as “large effect” [23]. For the Mann-Whitney *U* and Wilcoxon tests, the *r* formula was calculated based on the *z* scores. The measures between 0.1 and 0.3 were labeled “small effect,” between 0.3 and 0.5 as “moderate effect,” and above 0.5 as large effect” [23,24].

## Results

### Initial Observations

**Figure 1:**
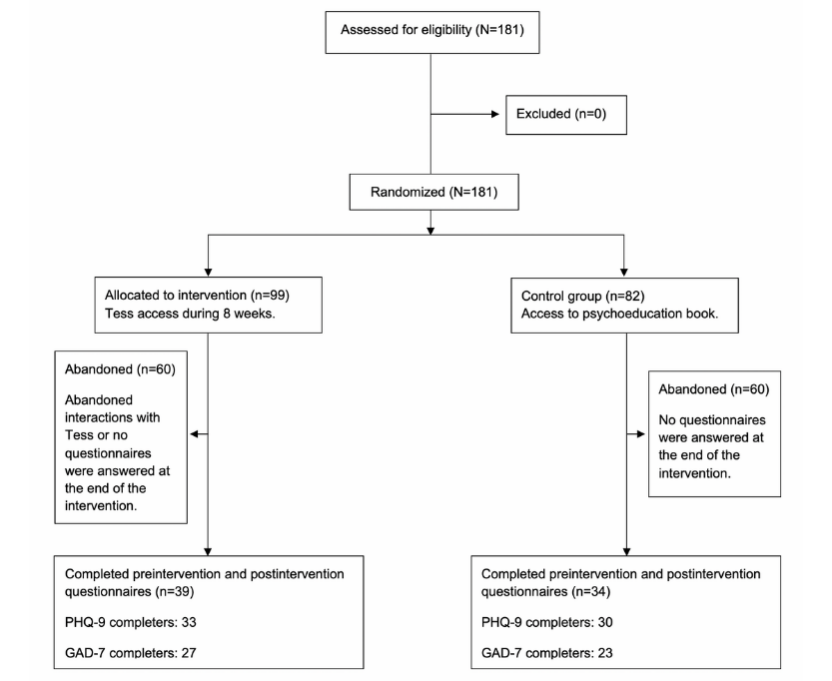
CONSORT flow diagram. GAD-7: Generalized Anxiety Disorder Scale-7, PHQ-9: Patient Health Questionnaire-9.

The initial sample consisted of 181 college students in Argentina, aged 18 to 33, with 158 (87.2%) identifying as female. Among the 181 students, 99 (55%) were randomized to the experimental condition and 82 (45%) to the control condition. Data at week 8 were provided by 39 out of the 99 (39%) participants in the experimental condition and 34 out of the 82 (41%) in the control group. Regarding data on the depressive symptoms, 33 (33%) participants in the experimental condition and 30 (37%) in the control condition provided data at week 8. Regarding data on anxiety symptoms, 27 (27%) participants in the experimental condition and 23 (28%) in the control condition provided data at week 8 (see Figure 1).

### Messages Exchanged

Regarding the participants’ engagement with Tess (39/99), after 8 weeks, there was an average of 472 exchanged messages (SD 249.52), where the minimum interaction level involved 162 messages and the maximum involved 1290. More specifically, an average of 116 (SD 73.87) of the exchanged messages were sent from the user to Tess.

#### Feedback

Feedback from most participants (25/39) at week 8 was coded as positive (ie, *Yes, you really understand me, Tess. Thanks for talking to me. My anxiety has decreased and I can confidently go outside again*.). A minor number of participants (7/39) provided ambivalent (ie, *Not much, but it’s ok, I am capable*) or negative (ie, *Sometimes I ask you something and you don’t specifically respond to what I asked.*). A one-factor ANOVA was applied to determine if feedback (positive, negative, or ambivalent) impacted the number of interactions that users had with Tess. Results showed that feedback from users was associated with the number of messages exchanged with Tess (*F*_2,36_=4.37; *P*=.02). Post hoc contrasts resulting from the Scheffé test showed statistically significant differences between those participants providing positive feedback and those providing negative feedback (*P*=.04); nevertheless, a higher number of messages exchanged with Tess was associated with positive feedback. No differences were observed between the participants providing ambivalent and positive feedback or ambivalent and negative feedback (See Table 1).

**Table 1:** Number of interactions per user feedback.

Variable	Positive, mean (SD)		Ambivalent, mean (SD)		Negative, mean (SD)		Statistical values	
							*F*	*P* value
Interactions	551.24 (52.54)		374.43 (73.11)		287.43 (23.35)		4.37	.02

### Potential Impact of Tess on Indicators of Depression and Anxiety

#### Baseline Characteristics

There were no statistically significant differences at the baseline in the anxiety (t_48_=.16; *P*=.9) and depression scores (*U*=451.50; *P*=.5) between the experimental and the control groups (See Table 2).

**Table 2:** Comparison of the average values and ranges for the anxiety and depression variables at the baseline and week 8 between the experimental and control groups.

Variable	Experimental group (n=39)	Control group (n=34)	Statistics	*P* value
Anxiety at baseline, mean (SD)	15.59 (5.30)^a^	15.35 (5.75)^b^	*t*=0.16	.90
Anxiety at week 8, mean (SD)	13.04 (7.12)^a^	16.26 (5.79)^b^	*t*=1.74	.09
Depression at baseline, middle range	33.32^c^	30.55^d^	*U*=451.50	.50
Depression at week 8, middle range	30.58^c^	33.57^d^	*U*=448.00	.48

^a^n=27.

^b^n=23.

^c^n=33.

^d^n=30.

#### Between-Group Differences

No statistically significant differences were observed between the experimental and the control groups in the average scores for anxiety (t_48_= 1.74; *P*=.09) or in the average ranges for depression (*U*=448.00; *P*=.48) at week 8 (See Table 2). Regarding the effect sizes, the mean scores for anxiety in the experimental group were lower than for the control group after 8 weeks and the effect size of the intervention was moderate (*d*=.5; 95% CI [-6.96 to.51]). For depressive symptoms, the experimental group reported a lower mean score than the control group and the effect size of the intervention was nonexistent (*r*=.09).

### Within-Group Differences

Within the experimental condition, a statistically significant decrease in the symptoms was observed from the baseline to week 8 for the anxiety scores (t_26_=2.15; *P*=.04); the control condition did not demonstrate any significant changes (t_22_=1.00; *P*=.33). Regarding depressive symptoms, no significant differences were found either in the experimental condition (*Z*=1.76; *P*=.08) or in the control condition (*Z*=.00; *P*>.99) (See Table 3).

**Table 3:** Comparison of the average values and ranges within groups for anxiety and depression variables from the baseline to week 8.

Variable and condition		Baseline	Week 8	Statistics	*P* value
**Anxiety, mean (SD)**					
	Experimental	15.59 (5.30)	13.04 (7.12)	*t*=2.15	.04
	Control	15.35 (5.75)	16.26 (5.79)	*t*=1.00	.33
**Depression, middle range**					
	Experimental	8.83	7.14	*Z=*1.76	.08
	Control	6.50	6.50	*Z=*0.00	.99

## Discussion

### Important Findings

The use of chatbots (ie, conversational agents) to address mental health conditions may contribute to the treatment of large populations and attend to the needs of those who do not have access to treatment. To the best of our knowledge, there are no studies on the use of chatbots for mental health in Latin America. This trial was intended to evaluate an AI-based chatbot (Tess) in a sample comprising Argentinian college students. The specific objectives were as follows: (1) understand the participant flow from recruitment to follow-up; (2) report aspects related to the usage patterns of Tess, such as the number of messages sent and exchanged; (3) examine participant feedback; and (4) compare the preliminary measures of depression and anxiety.

Regarding the usage patterns of Tess, there are three findings that support a satisfactory level of engagement. First, a considerable number of participants in the experimental (39/99, 39%) and control (34/82, 41%) conditions remained in the study throughout the 8-week study period. The completion rates found in the current study are better than that observed in most unpaid and unsupported Internet-based interventions for depression and anxiety, where 90% of the users withdraw after the first two sessions [25]. Furthermore, in studies using mobile apps, the follow-up completion rates were comparable (53%); the mean percentage of complete “adherers” was 36% for depression and 41% for anxiety [26]. When compared to a chatbot study for US college students, a lower attrition rate was reported (31% and 9% in the control and experimental conditions, respectively); however, this study compensated participants and the follow-up was at 2 weeks, making it difficult to compare the outcomes [6].

Second, participants in the experimental condition had exchanged a considerable number of messages with Tess (M 472; SD 249.52), and the mean number of messages sent from the user to Tess was 116 (SD 73.87). A previous study on the usage patterns of the depression modules of Tess showed a much lower average number (17.57) of messages sent to Tess by adult users [27]. It is possible that college, younger, and Latinx students are more willing to engage in conversations with chatbots than older populations in the United States. Two previous studies involving college students in the United States did not report the number of messages sent by the user to Tess [6,7]. Regarding the messages exchanged, Fulmer et al [7] reported a comparable number of total messages exchanged during a period of 4 weeks (M 286; SD 104.6), whereas the total number of messages exchanged in the current study was during a period of 8 weeks.

Third, feedback provided by those in the experimental condition was mostly positive (eg, *Yes, you really understand me, Tess. Thanks for talking to me.).* Among the participants offering negative feedback, there was a predominant dissatisfaction regarding the accuracy of some interventions ( eg, *Sometimes I ask you something and you don’t specifically respond to what I asked.*). Feedback is a key component for AI-based chatbots as it allows systems to tailor the dialogues to the user. Interestingly, the positive and negative feedbacks were associated with the number of messages exchanged. Users who reported higher satisfaction had the highest number of exchanged messages; it is possible that providing positive feedback could lead to better customization the intervention messages. This finding is relevant as it supports the need to collect user feedback for achieving optimal levels of customization and increasing engagement that could lead to higher intervention doses.

Regarding the impact of Tess on anxiety and depressive symptoms, no statistically significant differences were found between groups. Interestingly, when comparing within-group scores, the experimental group showed a significant decrease in anxiety symptoms after 8 weeks of intervention and a near-significant trend (*P*=0.07) for depressive symptoms. Analyzing the effect sizes showed that Tess had a moderate effect on anxiety and no effect on depression in the experimental group. These outcomes were unexpected given that previous studies using Tess [7] and another conversational agent called Woebot [6] reported significant reductions in anxiety and depressive symptoms; both studies used a similar control group (a psychoeducation book). Moreover, in the current study, depression was measured using PHQ-9 as a categorical and ordinal variable, whereas Fulmmer et al [7] and Fitzpatrick et al [6] used it as a continuous measure.

The lack of between-group differences could be explained by several factors. First, the current study was underpowered. Second, although the findings of the current study were not statistically significant, the direction of the change observed for anxiety and depression was as expected; therefore, it is possible that low-intensity interventions delivered via chatbots may require a higher dose to yield a between-group effect when delivered to Argentinian students. Third, Tess provides many conversations based on different theoretical approaches, and this may have resulted in less therapeutic power. However, Fulmer et al [7] observed significant effect using similar conversations. Fourth, it is possible that during adaptation of the dialogues from English to Spanish, the quality of the intervention may have been reduced.

### Limitations and Future Directions

This pilot study has several limitations. First, the current analysis was conducted with intervention completers; therefore, future studies with larger samples (including completer and intent-to-treat analyses) are needed. Second, only college students from a specific region in Argentina were included in this study, and the socioeconomic aspects of the sample were not assessed; thus, the inclusion of a more diversified sample is suggested. Third, there was a high dropout rate throughout the 8-week period. This is congruent with the findings reported by most studies that use technology-based intervention (see “The Law of Attrition”) [28]. A high dropout rate may be due to the limited capacity of most digital interventions to capture the attention and motivation of users. Additionally, high dropout rates in studies with digital interventions were linked to the fact that as access is easy, a lower level of commitment is required from the user to enroll in the study compared to traditional face-to-face interventions. Fourth, as most participants who remained until completion of the study were female, male participation was scarce. Fifth, the control group had access to a psychoeducation book, and there was no information on whether they read it. As chatbot research is in its initial stages, further studies could benefit from offering waitlists rather than self-help books. Although offering a waitlist could present an ethical dilemma, this would mitigate the potential effects of not having an intervention if short-term studies are conducted.

Future chatbot studies may benefit from designing chatbots with more conversations based on a specific therapeutic approach rather than using a few conversations from several approaches. Additionally, analyzing the impact of chatbots as adjuncts to face-to-face psychotherapy and comparing these interventions with face-to-face psychotherapy alone would yield important insights regarding the advancement of research on chatbots for mental health. Finally, simple randomization was used in this study; future studies may consider using unequal randomization (2:1) so that more participants enter the experimental group or a stratified randomization procedure so that participants with similar characteristics can be assigned equally to the experimental and control groups**.**

### Conclusions

Students spent a considerable amount of time exchanging messages with Tess and positive feedback was associated with higher numbers of messages exchanges. The initial results showed preliminary evidence regarding the effectiveness of Tess in addressing anxiety symptoms, but there was no significant effect on depressive symptoms in Argentinian college students. Given the high prevalence of anxiety and depression in Argentinian college students [1] and the need to expand mental health care access, developing affordable strategies such as chatbots may become effective tools to address these needs. AI-based chatbots have the ability to reach higher levels of customization and may thus be of service to educational and mental health care centers aiming to deliver interventions to targeted users that are accessible at any time without geographical restrictions. Additionally, chatbots may be used as standalone resources for those who have no access to treatment or as a complement to traditional treatments. Although the initial evidence on the efficacy of chatbots is promising, research on chatbots is still in its initial stages and presents several limitations. Thus, more robust evidence is needed.

## Appendix

Multimedia Appendix 1 CONSORT-eHEALTH checklist (v 1.6.1).

## Abbreviations

AI: artificial intelligence
BITs: behavioral intervention technologies
CBT: cognitive behavioral therapy
*DSM-IV*: *Diagnostic and Statistical Manual of Mental Disorders-IV*
GAD-7: Generalized Anxiety Disorder Scale-7
MYLO: Manage Your Life Online
PAHO: Pan American Health Organization
PHQ-9: Patient Health Questionnaire-9
